# Brain biomechanics during acute obstructive hydrocephalus in live rats

**DOI:** 10.1186/1743-8454-7-S1-S37

**Published:** 2010-12-15

**Authors:** Alexander V Shulyakov, Richard Buist, Stefan S Cenkowski, Marc R Del Bigio

**Affiliations:** 1 Department of Pathology, Faculty of Medicine, University of Manitoba, 715 McDermot Avenue, Winnipeg MB, R3E 3P5, Canada; 2 Manitoba Institute of Child Health, Winnipeg, Canada; 3 Department of Radiology, Faculty of Medicine University of Manitoba, 715 McDermot Avenue, Winnipeg MB, R3E 3P5, Canada; 4 Department of Biosystems Engineering, Faculty of Engineering, University of Manitoba, Canada

## Background

Predicted transmantle pressure gradients are not found in humans or in animals with hydrocephalus [1,2]. We hypothesize that pulsatile forces transmitted through incompressible cerebrospinal fluid (CSF) into viscoelastic brain tissue results in slowly accumulating strain that leads to subsequent ventricular enlargement. As a first step to proving this hypothesis, we have measured viscoelastic properties in living rat brain.

## Materials and methods

Young adult rats (age 56-70 days; n=18) had hydrocephalus induced by kaolin injection into the cisterna magna. Ventricles size, cerebral blood flow (CBF) before and after craniotomy was assessed by magnetic resonance imaging (MRI). At several time points after kaolin injection (on 3-4, 7-9 and 12-15 day) a craniotomy was performed and viscoelastic parameters (elastic modulus, brain creep and softening) were determined in live brain with intact dura using microindentation testing. Contact cortical CBF was also acquired using a laser Doppler device incorporated into the indentation sensor. Brain intraparenchymal pressure (IPP) was measured simultaneously.

## Results

MRI showed progressive ventricular enlargement after kaolin injection. There was a significant increase of the cortical cerebral blood flow (measured by MRI arterial spin labelling) following craniotomy. Cortical CBF ascertained by laser Doppler did not change appreciably as hydrocephalus progressed, however it decreased up to 30% at the site of indentation testing. A 2-fold IPP increment was observed at days 3-4 and 7-9 relative to the normal value (8-10 mm Hg). Instrumented brain indentation with low loading force (0.07-0.09 N) and loading-unloading rate of 0.14-0.18 N/min revealed a decrease of brain elasticity 3-4 and 12-15 days after kaolin injection. Viscoelastic creep increased at 3-4 days and was double the normal value at 12-15 days. Brain softening on multicycle indentation was increased 7-9 days after kaolin injection.

## Conclusions

Living brain exhibits mechanical properties consistent with a viscoelastic nature. During the early development of hydrocephalus, the mechanical properties are modified at a time when overt histopathological changes would not be expected. The act of measuring the properties results in physiological changes, which must be considered as this series of experiments progresses.
